# Virome of *Camellia japonica*: Discovery of and Molecular Characterization of New Viruses of Different Taxa in Camellias

**DOI:** 10.3389/fmicb.2020.00945

**Published:** 2020-05-15

**Authors:** Song Zhang, Liu Yang, Lisha Ma, Xin Tian, Ruhui Li, Changyong Zhou, Mengji Cao

**Affiliations:** ^1^National Citrus Engineering and Technology Research Center, Citrus Research Institute, Southwest University, Chongqing, China; ^2^State Cultivation Base of Crop Stress Biology for Southern Mountainous Land, Academy of Agricultural Sciences, Southwest University, Chongqing, China; ^3^USDA-ARS, National Germplasm Resources Laboratory, Beltsville, MD, United States

**Keywords:** *Camellia japonica*, next-generation sequencing, virome, new viruses, RT-PCR detection, phylogenetic analysis

## Abstract

Many species of the genus *Camellia* are native to China, and several species such as *C. japonica* have been cultivated as garden plants for over 1,000 years. Virus-like symptoms have been recorded for years. In this study, *C. japonica* plants with various leaf symptoms were observed in Jiangxi and Chongqing provinces. The species composition of potential viruses in the symptomatic plants was analyzed by next-generation sequencing of six libraries prepared from total RNAs of specimens from 10 trees. Five new viruses were discovered, and their genome sequences were determined. These viruses were tentatively named Camellia chlorotic ringspot viruses (CaCRSVs), Camellia yellow ringspot virus (CaYRSV), Camellia-associated badnavirus (CaBaV), and Camellia-associated marafivirus (CaMaV) based on comprehensive analyses. Among these viruses, CaYRSV, CaBaV, and CaMaV share similar genome organizations and clear sequence homology with known viruses in databases and could potentially be classified as new species of the genera *Badnavirus, Idaeovirus*, and *Marafivirus*, respectively. CaCRSVs comprise two distinct viruses, and each likely contains five genomic RNA segments that were found to be distantly related to viral RNAs of members in the genus *Emaravirus* (family *Fimoviridae*). The RNAs of CaCRSVs show conserved terminal sequences that differ markedly from those of emaraviral RNAs. These data, together with the phylogenetic analysis, suggest that the evolutionary status of CaCRSVs may represent a novel genus in the family *Fimoviridae*. In addition, two known viruses (geminivirus and blunervirus) and a mass of betaflexiviruses existing as heterogeneous mixtures were detected, and their roles in symptom formation were studied. Collectively, the information of the viral species and detection protocols that were developed can serve as a basis for better management of these viruses. Distinguishing the virus-related symptoms from genetic characteristics of *C. japonica* is also significant for breeding efforts.

## Introduction

*Camellia* spp. of the family *Theaceae* are economically important group of perennial evergreen flowering plants (Gao, 2005). This genus of approximately 280 species are native to East and Southeast Asia (Meegahakumbura et al., 2018). Most (238 species) are naturally distributed in China (http://www.iplant.cn/info/Camellia?t=z). *C. sinensis* is planted to produce popular tea beverages, while *C. japonica* (common camellia) is a well-known ornamental shrub. *C. japonica* and its hybrids are well-known ornamentals since they have large flowers of various colors and shapes, long and varied blossoming seasons and different growth habitats (Mondal, 2011). Ornamental camellias (chahua in Chinese) have been grown in China since Three Kingdoms Period (AD 220–265) and are the symbolic flowers of Chongqing and Yunnan. The common camellia was introduced to Japan where it was named Tsubaki to be distinguished from Sazannka (*C. sasanqua*), the Japanese camellia, over 1,000 years ago (Wu Y. et al., 2015). The ornamental camellias were brought to Europe and Americas in late 1870's (Bartholomew, 1986), and are now popular flowering and landscaping shrubs in many regions with mild climate in the world (Mondal, 2011). Additionally, camellias contain many bioactive compounds such as tea saponins with surface-active properties and pharmacological activities (Zhao et al., 2011).

Both biotic (fungal, bacterial, and viral diseases) and abiotic stresses affect ornamental camellias (Dickens and Cook, 1989; Taylor and Long, 2000; Zhang et al., 2014). Fungal pathogens such as those of leaf spots and gray blight are the primary concerns of camellias in China (Zhang et al., 2012; Yang S. et al., 2019), while viruses have not been well-studied, regardless of being suspected to be associated with some leaf-related diseases for decades (Milbrath and McWhorter, 1946; Gailhofer et al., 1988). The virus-like symptoms such as foliar mottle, mosaic, ringspots as well as foliar and flower variegations have been observed on *C. japonica* (Milbrath and McWhorter, 1946; Hildebrand, 1954; Ahlawat and Sardar, 1973; Gailhofer et al., 1988). These viral diseases could easily be transmitted across generations and spread between different regions by vegetative propagation (cutting and grafting) commonly used by commercial companies and individuals (Inouye, 1982). The variegation caused by the viruses may be confused with genetic variegation, which is valuable horticultural trait (Valverde et al., 2012). The putative viruses were associated with some viral diseases by biological and morphological studies (Plakidas, 1954; Inouye and Inouye, 1975; Hiruki, 1985; Gailhofer et al., 1988). With the application of next-generation sequencing (NGS) techniques, several new viruses have been recently identified from camellias with different symptoms (Hao et al., 2018; Zhang et al., 2018; Liu H. et al., 2019). However, the studies of the potentially implicated viruses are still inadequate, especially with respect to genome information.

Replication cycle of viruses with both RNA and DNA genome and viroids has an mRNA transcript and/or RNA replication stage. The enhancement during replication of their genomes inevitably increases the generation of double-stranded RNA (dsRNA), which can be degraded to the virus- or viroid-small RNAs (sRNAs) by the RNA silencing of the host plants (Ding, 2010). Therefore, the sequencing of plant total RNAs or sRNAs of the hosts is able to capture almost all sequence information of viruses and viroids in tested plant tissues (Wu Q. et al., 2015). The two sequencing techniques have some advantages and shortcomings (Pecman et al., 2017), and a combined utilization is also used in the virome analyses (Cao et al., 2019). Here, we used ribosome RNA-depleted RNA sequencing to analyze *C. japonica* plants displaying various symptoms, which allowed the identification of five new viruses, with several of them being exclusively associated with one distinct symptom based on comparative analysis.

## Materials and Methods

### Plant Materials

**Figure 1:**
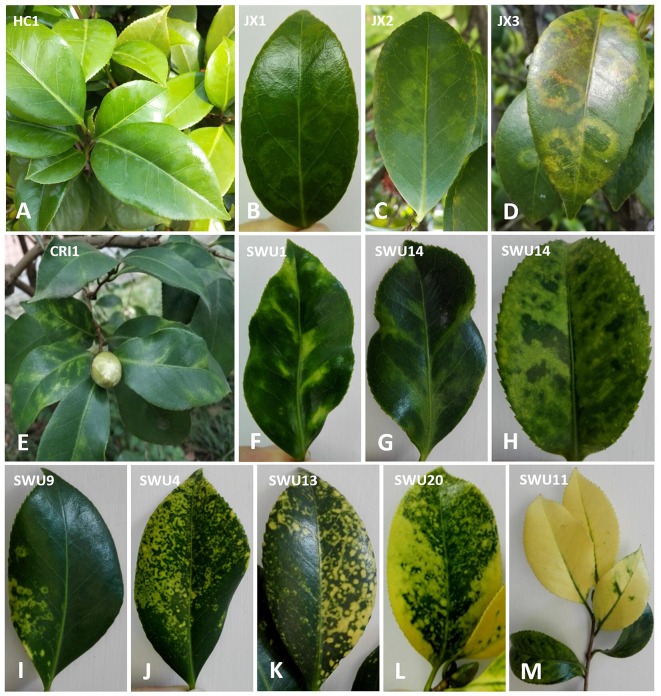
Symptom observation of *Camellia japonica* leaves. **(A)** No obvious symptoms. **(B–D)** Chlorotic ringspot. **(E–G)** Malformation and mosaic. **(H)** Chlorotic mottle. **(I–L)** Yellow ringspot, yellow spot, yellow mottling, and yellowing. **(M)** Yellowing.

Leaf samples of ten *C. japonica* trees, nine from Chongqing province (HC1, CRI1, CRI2, SWU1, SWU4, SWU11, SWU13, SWU14, and SWU20) and one from Jiangxi province (JX1), were collected during 2016–2018 (Table S1). According to similar leaf symptoms, these samples were divided into six groups designated as SC-HC (HC1; non-symptomatic), SC-JX (JX1; chlorotic ringspot), SC-CRI (CRI1, CRI2; malformation and mosaic), SC-L16 (SWU11; yellowing), SC-L17 (SWU1, SWU14; mosaic and chlorotic mottle), and SC-L18 (SWU4, SWU13, SWU20; yellow ringspot, yellow spot, yellow mottle, and yellowing) (Figure 1). The six sample groups were each tested by NGS. Ten grams of leaf tissues from each sample group was ground in liquid nitrogen to fine powder. One gram of the powder was used for RNA extraction, and the rest powder was stored at −80°C for future use.

### RNA Extraction, NGS, and Data Processing

Total RNA was extracted using the EASY spin Plus Complex Plant RNA Kit (Aidlab, China), and then tested using the Nanodrop (Thermo Fisher Scientific, USA), Qubit 3.0 (Invitrogen, USA), and Agilent2100 (plant RNA Nano Chip, Agilent, USA) for purity, concentration, and integrity, respectively. After the removal of ribosome RNA by the Ribo-Zero Magnetic Kit (Epicenter, USA), the libraries were built using a TruSeq RNA Sample Prep Kit (Illumina, USA). An Illumina HiSeq X-ten platform (Illumina) set with length of 150-bp pair-end reads was then used for sequencing (Mega Genomics, China). Sequences of adaptor and low-quality trait were trimmed from raw reads, and the rest reads were mapped to the genome sequences of common tea (*C. sinensis*) (Wei et al., 2018), using the CLC Genomic Workbench 9.5 (Qiagen, USA). The reads with sequence similarities of >60% to the tea genome sequences were eliminated to reduce interference of the host background, and the remaining unique reads were *de novo* assembled using the Trinity program (Grabherr et al., 2013). The resulted contigs were subjected to BLASTx and BLASTn searches against viral (taxid:10239) and viroidal (taxid:2559587) sequences of local datasets retrieved from the National Center for Biotechnology Information (NCBI) databanks. These processes allowed the identification of the contigs with viral sequence attributes.

### Recovery of Viral Genomes

A set of specific primers based on the viral contig sequences were designed using the Primer Premier 5 (Premier Biosoft, USA) to amplify overlapping fragments of each of the new *C. japonica* viruses (Figure S1). The primers are listed in Table S2. One-step reverse transcription-PCR (RT-PCR) assay was carried out using the PrimeScript One-Step RT-PCR Kit (Takara, Japan). Rapid amplification of cDNA ends-PCR (RACE-PCR) assay was conducted using the GeneRacer Core Kit (Invitrogen, USA). PCR assay was done with the 2 × Taq Master Mix Kit (Quick Load) (Novoprotein, China). The PCR amplicons were purified by the Gel Extraction Kit (Biomega, USA) and cloned into the pEASY-T1 Vector (TransGen, China). Sequence of each amplicon was determined from both directions of five clones by a biotechnology company (Tsingke, China). The full-length genome of each virus was assembled from all amplicons of the virus using the *de novo* assembly algorithm in SeqMan (DNAStar, USA).

### Sequence Analysis and Read Assembly

Viral genome organizations were studied using the ORF finder (https://www.ncbi.nlm.nih.gov/orffinder) and the Conserved Domain Database (CDD) (https://www.ncbi.nlm.nih.gov/Structure/cdd/wrpsb.cgi) websites in NCBI for opening reading frames (ORF) with a length >300-nucleotide (nt) and conserved amino acid (aa) domains with an e-value <0.05, respectively. The DRNApred (http://biomine.cs.vcu.edu/servers/DRNApred/), TMHMM (http://www.cbs.dtu.dk/services/TMHMM/), and PROMALS3D (http://prodata.swmed.edu/promals3d/promals3d.php) were used to predict DNA-binding sites, transmembrane (TM) domains, and secondary structures of viral proteins inferred from ORFs, respectively. Nucleotide or aa sequence alignment and comparison were performed using the CLC Genomic Workbench 9.5.

A total of 9.54–11.35 G trimmed reads of six datasets were individually generated from the six independent leaf sample sets after a pipeline of data processing (Table S1). Subsequently, the reads (91.77–95.99%) mapped to the tea genomes as references were removed. Finally, assembly of the remaining 4.01–8.23% unique reads generated 13,583–34,687 contigs ranged from 200 to 8,789 nt in size. BLASTx analysis of the contigs using default parameters revealed the virus-related contigs that were homologous to several different taxa of viruses, including badnavirus, betaflexiviruses, blunervirus, emaravirus, geminivirus, idaeovirus, and marafivirus.

### Phylogenetic Analysis

The genome (nt) or protein (aa) sequences of each of the new viruses identified by NGS and its closely related viruses retrieved from NCBI databases were aligned by the CLC Genomic Workbench 9.5. Phylogenic trees were constructed by the MEGA 7.0 (Kumar et al., 2016) using a neighbor-joining method with layouts of Jones-Taylor-Thornton (aa) or Maximum Composite Likelihood (nt, transitions + transversions) model substitution, complete deletion treatment of gaps, and 1,000 bootstrap replications.

### Virome and PCR Analysis

Viral species of each sample group, RNA reads of each virus, and the proportion of viral reads in total reads were statistically analyzed. Venn diagrams were drawn using a website tool (http://bioinformatics.psb.ugent.be/webtools/Venn/). The copy number (average coverage) of viral RNA was calculated by multiplying the number of viral reads by the average length of total reads (about 150 nt) and dividing that result by the length of viral RNA.

The occurrence of viruses in 37 *C. japonica* trees (including 9 trees sequenced by NGS) from the Jiangxi and Chongqing provinces was investigated using the PCR or RT-PCR protocols (Cao et al., 2019), specific primers designed in previous studies (Hao et al., 2018; Zhang et al., 2018), and the primers designed by the DNAMAN 7 (Lynnon Biosoft, Canada) in this study (Table S2).

## Results

### Identification of Viruses Infecting the Camellias

Among all the viral contigs, the betaflexivirus-related contigs accounted for 68% (59 of 87), which were detected in all the six sample groups (Table S1). Thus, these sequences were numerous and complicated, and the analysis below suggested that they were not associated with any observed symptoms. Therefore, the sequences of this taxon were not emphasized in the present work. We will focus on the molecular characterization of the five newly identified viruses related to badnavirus, emaravirus, idaeovirus, and marafivirus.

### Two Known Camellia Viruses

The geminivirus- and blunervirus-related contigs shared more than 98% nt sequence identity with Camellia chlorotic dwarf-associated virus (CaCDaV; Zhang et al., 2018) and tea plant necrotic ring blotch virus (TPNRBV; Hao et al., 2018), respectively. These results confirmed the presence of the two viruses in *C. japonica*.

### A New Monopartite Positive-Stranded RNA Virus

**Figure 2:**
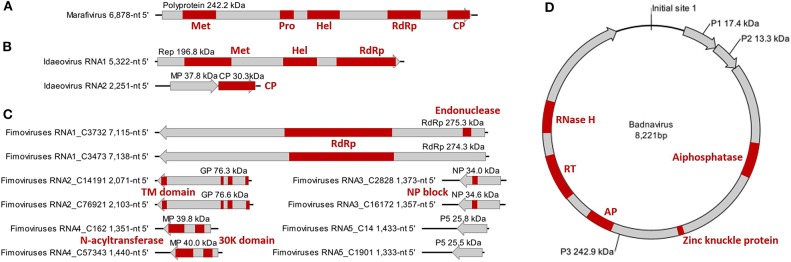
Genome and protein features of Camellia-associated marafivirus (CaMaV) **(A)** and badnavirus (CaBaV) **(D)**, and Camellia yellow ringspot virus (CaYRSV, the idaeovirus) **(B)** and chlorotic ringspot viruses (CaCRSVs, the fimoviruses) **(C)**, with conserved aa domains or motifs indicated by red boxes. Met, methyltransferase; Pro, protease; Hel, helicase; RdRp, RNA-dependent RNA polymerase; CP, coat protein; Rep, replicase; MP, movement protein; GP, glycoprotein; TM, transmembrane; NP, nucleocapsid protein; AP, aspartate protease; RT, reverse transcriptase; RNase H, ribonuclease H.

**Figure 3:**
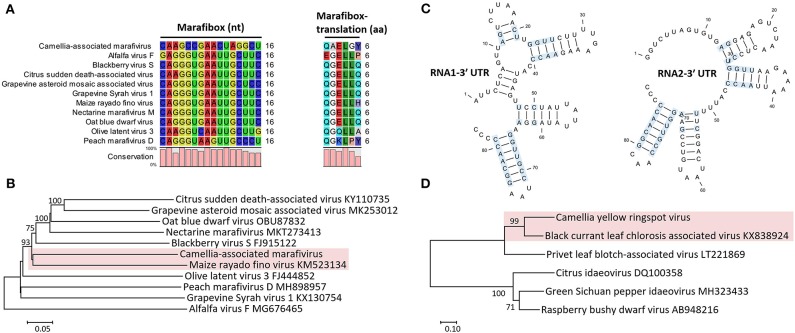
**(A)** Multiple sequence alignment of Camellia-associated marafivirus (CaMaV) and representative marafiviruses at the marafibox region. **(B)** Phylogenetic analysis of CaMaV and the marafiviruses based on the whole genome sequences; bootstrap values (1,000 replications) under 50% are pruned. The CaMaV and its closest relative are indicated by the red background. **(C)** Phylogenetic relationships inferred from the conserved coat protein (CP) amino acid sequences of Camellia yellow ringspot virus (CaYRSV, the idaeovirus) and representative idaeoviruses, with CaYRSV and the closest relative backgrounded by red. Significant bootstrap values that were greater than 50% (1,000 replications) are shown. **(D)** Secondary structure of 3′ untranslated regions (3′ UTR) of genomic RNAs of CaYRSV. The blue background highlights the identical nucleotides between the stem-loops of RNA1 and RNA2.

The monocistronic genome of the marafivirus-related virus (Figure 2A) is 6,878 nt long, excluding the poly (A) tails. It had the highest nt sequence identity (55.8%) to grapevine asteroid mosaic-associated virus (GAMaV, MK253012) (Vargas-Asencio et al., 2017). The 5′ untranslated region (5′ UTR, 140 nt) and 3′ UTR (123 nt) shared the highest 40 and 71.3% nt sequence identities with nectarine marafivirus M (NeVM, KT273413) (Villamor et al., 2016) and Citrus sudden death-associated virus (KY110735) (Maccheroni et al., 2005), respectively. Its genome organization is typical of marafiviruses, containing a single ORF (Igori et al., 2017). This ORF (nt 141–6,755) encodes a large putative polyprotein (2,204 aa, 242.2 kDa) consisting of a replication-associated polyprotein (RP) with a methyltransferase (Met, pfam01660, aa 145–426), a protease (Pro, cl05113, aa 840–939), a helicase (Hel, pfam01443, aa 1,030–1,262) and an RNA-dependent RNA polymerase (RdRp, cl03049, aa 1,600–1,836), and a coat protein (CP, cl03052, aa 2,029–2,188). The RP and the CP were most related to the NeVM (54.7% aa sequence identity) and the GAMaV (60.3% nt and 58.6% aa sequence identity), respectively. A 16-nt conserved nucleotide sequence stretch called “marafibox” [CA(G/A)GGUGAAUUGCUUC] (Izadpanah et al., 2002) was not found, but the RP amino acid sequences associated with the “marafibox” were partially identical to those of marafiviruses (Figure 3A).

Phylogenetic relationships constructed using the whole-genome sequences placed the marafivirus-related virus and maize rayado fino virus (KM523134) (Hammond and Ramirez, 2001) in a subgroup in the marafivirus group (Figure 3B). The results for the marafivirus-related virus satisfy the species demarcation criteria (<80% identical at whole genome sequence and <90% identical at coat protein sequence) of the genus *Marafivirus* (Dreher et al., 2011). Thus, this virus should be a new species of the genus.

### A New Bipartite Positive-Stranded RNA Virus

The genome of the idaeovirus-related virus is composed of two genomic RNA components (RNA1 and RNA2) (Figure 2B). They shared the greatest nt sequence identities of 66.5 and 66.4% with RNA1 (KY399998) and RNA2 (KY399999) of black currant leaf chlorosis-associated virus (BCLCaV), respectively (James and Phelan, 2017). Like other idaeoviruses (Navarro et al., 2017), both RNAs start with a 5′ end tetranucleotide (AUAU), and end with 3′-terminal four stem-loop structures and cytidine (C) repeats (Figure 3C).

RNA1 (5,322-nt, ORF in nt 52–5,238) encodes a putative replicase (Rep) protein (1,728 aa and 196.8 kDa) consisting of conserved Met (pfam01660, aa 195–543), Hel (pfam01443 aa 895–1,146), and RdRp (pfam00978, aa 1,276–1,709) domains. The Rep was most homologous to the BCLCaV (74.2% aa sequence identity). The 5′ and 3′ UTRs shared the highest nt sequence identities with privet leaf blotch-associated virus (54.6%) (Navarro et al., 2017) and the BCLCaV (53.6%), respectively.

RNA2 (2,251-nt) contains two ORFs with one nucleotide overlap that encode a putative movement protein (MP, 343 aa and 37.8 kDa) at nt 319–1,347 and a putative CP (cl05884, 270 aa and 30.3 kDa) at nt 1,350–2,159. It was most related to the BCLCaV at 5′ UTR, 3′ UTR, MP gene, and CP gene, for which the sequence identities shared were 54.5% (nt), 52.6% (nt), 69% (aa), and 71.2% (aa), respectively. The phylogenetic analysis based on the CP gene also suggested the closest relationship with the BCLCaV (Figure 3D). Given the differences with the BCLCaV in sequence and host, the idaeovirus-related virus was deemed as a putative new species in the genus *Idaeovirus*.

### Two Novel Multipartite Negative-Stranded RNA Viruses Associated With Emaraviruses

Ten contigs related to the genus *Emaravirus* (family *Fimoviridae*) were identified in the JX1 tree. The complete sequences of these RNA fragments were determined by Sanger sequencing (Figure S1). These ten viral RNAs could be divided into two groups according to significant aa sequence differences (25.2–57.3%) (Table S3) and difference of the RNA copy numbers [3-digit vs 2-digit (4 out of 5)] between the two groups (Table S4). Each group harbors five RNAs that encode core proteins with similarities to those of the emaraviruses, suggesting the existence of two putative fimoviruses.

**Figure 4:**
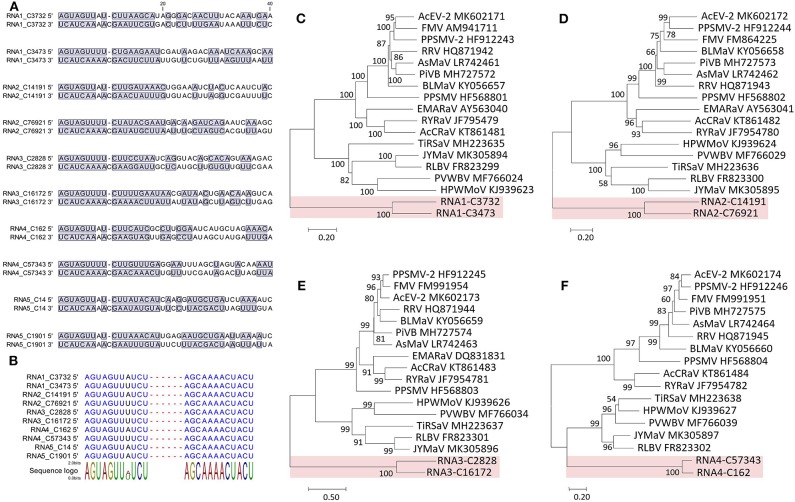
Sequence comparison of the 5′ and 3′ genomic ends of Camellia chlorotic ringspot viruses (CaCRSVs, the fimoviruses) and the complementary nucleotides are indicated with a blue background **(A)**. Multiple sequence alignment of both genomic termini of viral RNAs of CaCRSVs, and the identical nucleotides are displayed in blue **(B)**. Evolutionary analysis of CaCRSVs and representative emaraviruses based on amino acid sequences of RdRp **(C)**, GP **(D)**, NP **(E)**, and MP **(F)** genes. The genes of CaCRSVs are indicated by the red background. Bootstrap values (1,000 replications) below 50% are not shown.

The 5′ and 3′ ends were highly complementary in all the RNAs (Figure 4A), but a C residue that invariably occurs at the 10th nt position of the 3′ end (counting from 3′ to 5′) was exceeding and non-complementary, which was different from the emaraviruses (Mielke and Muehlbach, 2007; Mielke-Ehret and Mühlbach, 2012). For all of the RNAs, the 5′ and 3′ termini were conserved in the 11-nt (AGUAGUUWUCU, W = A/U) and 12-nt (AGCAAAACUACU), respectively (Figure 4B). The terminal consensuses were unique since the emaraviruses had a 13-nt consensus at each of the termini (5′- AGUAGUGUUCUCC……GGAGUUCACUACU-3′, the identical nt between the putative fimoviruses and the emaraviruses were underlined) (Mielke and Muehlbach, 2007). Furthermore, the GC content of 30% at the termini of the two putative fimoviruses was lower than average of 46% for the emaraviruses.

An AUG-initiated ORF that encodes a hypothetical protein was predicted for each of the ten RNA segments named RNA1-C3732, RNA1-C3473, RNA2-C14191, RNA2-C76921, RNA3-C2828, RNA3-C16172, RNA4-C162, RNA4-C57343, RNA5-C14, and RNA5-C1901 (Figure 2C). The lengths of the genomic 5′ UTRs (42- to 741-nt) and 3′ UTRs (38- to 154-nt) are variable, similar to those reported for the emaraviruses (Yang C. et al., 2019). All of the putative proteins were also related to the emaraviruses based on BLASTp analysis.

RNA1-C3732 of 7,115 nt and RNA1-C3473 of 7,138 nt contain an ORF (nt 7,050–88 for C3732; nt 7,138–92 for C3473) that encodes a putative RdRp (2,320 aa and 275.3 kDa for C3732; 2,325 aa and 274.3 kDa for C3473). The CDD search revealed a Bunya_RdRp superfamily domain (cl20265) for both the proteins and an endonuclease domain (cl20011) for the protein of the C3473 (Figure 2C). The aa sequences of these two proteins were 58.1% identical to one another, while only 24.1–28% identical to that of RdRps of the emaraviruses (Table S3).

RNA2-C14191 and RNA2-C76921 are 2,071 nt and 2,103 nt long, respectively. Their ORFs (nt 2,002–47 for C14191; nt 2,025–43 for C76921) encode putative glycoproteins (GP) of 651 aa (76.3 kDa) and 660 aa (76.6 kDa), respectively. The GP aa sequences were 16.7–20.8% identical to those of the emaraviruses and 44.3% with one another (Table S3). Three N-terminal TM domains and a C-terminal TM domain that were akin to those of the emaraviruses (Yang C. et al., 2019) were predicted in each of the GPs (Figure 2C).

RNA3-C2828 and RNA3-C16172 are 1,373 and 1,357 nt, respectively. They contain one ORF (nt 1,258–371 for C2828; nt 1,241–342 for C16172) that was predicted to encode putative nucleocapsid proteins (NP) of 295 aa (34 kDa) for C2828 and 299 aa (34.6 kDa) for C16172. An amino acid block, NXL-GXEX6PXE, conserved in the emaraviruses was identified in the two putative fimoviruses (Figure 2C), whereas another conserved block (NX2SX5A) was absent (Elbeaino et al., 2009). The NPs of the two putative fimoviruses shared very limited aa sequence identities of 11.6–18.3% with those of the emaraviruses and 43.7% with one another (Table S3).

RNA4-C162 (1,351 nt) and RNA4-C57343 (1,440 nt) have one ORF at nt 1,197–175 and nt 1,351–326, respectively. This ORF encode a putative movement protein (MP) of 340 aa (39.8 kDa) for C162 or 341 aa (40 kDa) for C57343. The 30K-MP structural signatures, including a putative catalytic Asp (D) residue and a series of alpha-helixes and beta-strands, were present based on the secondary structure analysis (Figure S2; Yu et al., 2013). The 30K domain was followed by an N-acyltransferase superfamily (cl17182) (Figure 2C). The identities of the amino acid sequences of the MP were 11.8–21.7% between the two putative fimoviruses and the emaraviruses and 74.8% between the two putative fimoviruses (Table S3).

RNA5-C14 of 1,433 nt and RNA5-C1901 of 1,333 nt contain a single ORF (nt 1,395–742 for C14 and nt 1,295–642 for C1901) coding for putative proteins of the same size (217 aa) with molecular weight of 28.8 and 25.5 kDa, respectively. The two proteins shared 53.9% aa sequence identify with each other, and approximately 21% aa sequence identify with the putative protein encoded by RNA7 of high plains wheat mosaic virus (KJ939630) (Table S3; Tatineni et al., 2014). These protein homologs may play similar roles fighting against the RNA silencing defenses of the host (Gupta et al., 2018, 2019).

The proteins encoded by RNA1–RNA4 were considered as the core elements because they are conserved for all assigned and unclassified members of the genus *Emaravirus* in the family *Fimoviridae* (Elbeaino et al., 2018). Phylogenetic analyses using the aa sequences of three of these proteins (RNA1–RNA3) all placed the two putative fimoviruses in a cluster distinct from the two subclusters formed by the emaraviruses (Figures 4C–F), supporting that they are new members of the family with an extraordinary evolutionary path.

Based on the facts that the two putative fimoviruses have moderate aa sequence identities (<74.8%) shared between them, unique termini at the two ends of the all five RNAs, low aa sequence identities (<28%) of their deduced proteins with the emaraviruses, and the evolutionary status representing a special clade of the family *Fimoviridae*, we propose these viruses as two putative species of a new taxon (genus) in the recently established family *Fimoviridae* (Elbeaino et al., 2018).

### A New Double-Stranded Circular DNA Virus

The badnavirus-related virus has a circular DNA genome of 8,221 bp, which contains three ORFs on the plus strand (Figure 2D). The RNA reads mapping analysis (Figure S3) showed that the mapped reads in the viral genome were overlapping and continuous, suggesting the episomal form of the virus rather than fragments integrate into host genomes. Multiple sequence comparisons at the whole genome level showed 31.3–37.4% nt identities between this virus and classified members of the genus *Badnavirus*. The genome contains the tRNAmet-binding site (TGGTATCAGAGCTTCGGC, nt 1–18), the TATA boxes (nt 109–112, 393–396, and 398–401), and the polyadenylation signal (AATAAA, nt 8,139–8,144), which resembled those of badnaviruses (Bouhida et al., 1993).

ORF1 (nt 421–870) encodes a putative protein P1 (149 aa, 17.4 kDa), which shared the highest aa sequence identity of 53.6% with the P1 of cacao swollen shoot Togo A virus (AJ781003) (Oro et al., 2012). A DUF1319 superfamily (cl06184) of unknown function that was possibly virion-associated was found in the P1 (Cheng et al., 1996).

ORF2 (nt 870–1,226) encodes a putative nucleic acid-binding protein, P2 (118 aa, 13.3 kDa) that had the highest aa sequence identity (34.3%) with Dioscorea bacilliform ES virus (KY827394) (Sukal et al., 2017). The P2 was predicted to have a DNA-binding region at aa 28–43 (Jacquot et al., 1996).

ORF3 (nt 1,223–7,777) encodes a putative polyprotein P3 (2,184 aa, 242.9 kDa). The P3 shared the highest aa sequence identity of 33.6% with that of Dioscorea bacilliform AL virus 2 (DBALV2, MH404155) (Sukal et al., 2020). The domains (Figure 2D) identified in the P3 include zinc knuckle protein (pfam00098, aa 999–1,016), aspartate protease (AP, cl11403, aa 1,295–1,389), reverse transcriptase (RT, cd01647, aa 1,511–1,694), and ribonuclease H (RNase H, cl14782, aa 1,793–1,921), which are typical of the genus *Badnavirus* (MacFarlane, 2011). In addition, a trimeric dUTP diphosphatase (cl00493, aa 505–640) was found in the P3.

**Figure 5:**
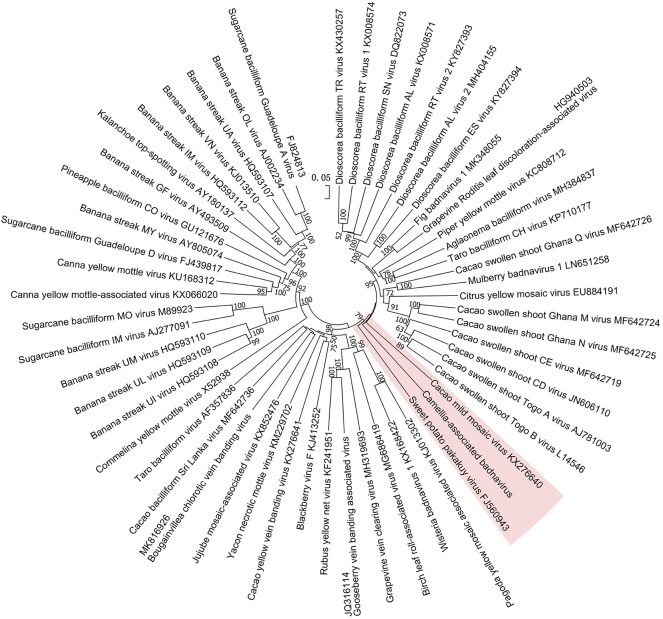
Phylogenetic analysis of full-genome sequences of Camellia-associated badnavirus (CaBaV) and representative badnaviruses. The CaBaV and its most associated viruses are indicated by the red background. Bootstrap values (1,000 replications) are shown only if they were above 50%.

The phylogenetic tree constructed by the whole-genome sequences of the badnavirus-related virus and representative badnaviruses grouped it with cacao mild mosaic virus (KX276640) (Chingandu et al., 2017) and sweet potato pakakuy virus (FJ560943) (Kreuze et al., 2009) in the same subcluster (Figure 5). Despite the close relationship with badnaviruses, the highest nt sequence identity of 66% shared between the virus and badnaviruses (DBALV2) at the regions combined with the RT and RNase H domains did not reach the species demarcation level (80%) of the genus *Badnavirus* (Geering and Hull, 2011), suggesting that the virus should be considered a new, distinct badnavirus species.

### Virome and Symptomatology Analysis

**Figure 6:**
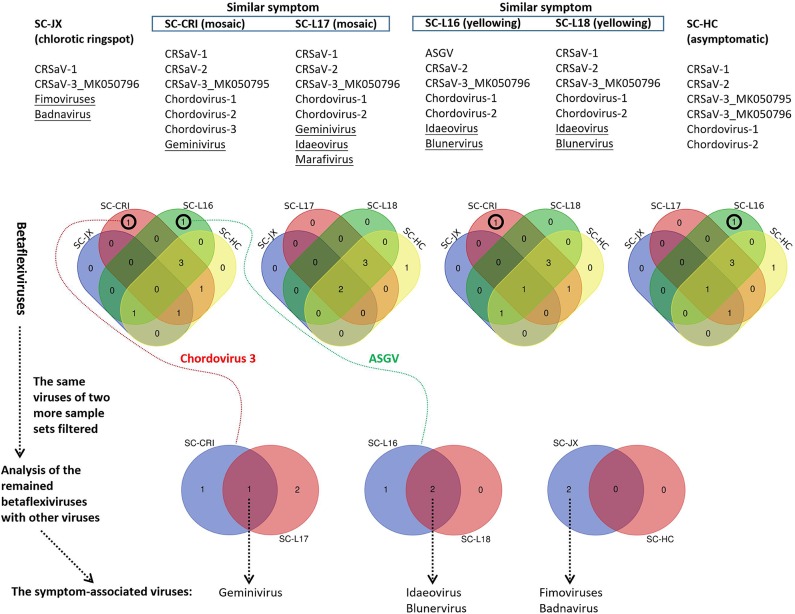
Comparative analysis of viruses in six sample sets (SC-HC, SC-JX, SC-CRI, SC-L16, SC-L17, and SC-L18), using Venn diagrams. At first, the similar betaflexiviruses of two more sample sets showing distinct symptoms were removed. Then, the same viruses of two sample sets of similar symptoms were considered to be symptom-related. ASGV (apple stem grooving virus), CRSaV-1, -2, and -3 (Camellia ringspot associated virus 1, 2, and 3), chordovirus-1, -2, and -3 (the three potential new chordoviruses identified in this study).

The 59 betaflexivirus-like contigs, the sequences and taxonomy of which will not be discussed in this study, were categorized according to 80% (considered as single putative virus as contigs shared >80% nt sequence identity). Then, based on the BLASTn identity of each putative virus (the contigs) shared with its closet relative available in databases, they were identified as apple stem grooving virus (ASGV, KR106996; 1 contigs, 78.6%), Camellia ringspot associated virus 1 (CRSaV-1, MK050792; 8 contigs, 79–96%), CRSaV-2 (MK050793 and MK050794; 16 contigs, 80–94%), CRSaV-3_MK050795 (3 contigs, 88–97%), CRSaV-3_MK050796 (6 contigs, 92–99%), and three potential new chordoviruses indicated by chordovirus-1 (8 contigs, 68–72%), -2 (16 contigs, 68–76%) and -3 (1 contigs, 66.6%). The comparative analysis (Figure 6) suggested that the betaflexiviruses and the marafivirus had minor effects on the development of the different symptoms observed on *C. japonica* plants.

PCR and RT-PCR using specific primers showed that CCaDaV was found in 18 camellias exhibiting mosaic and/or malformation, the idaeovirus was detected in 13 camellias with yellowing, yellow spots or yellow ringspots, the fimoviruses were present in 8 camellias of chlorotic ringspots, while some of the symptomatic camellias might be only infected by one of these three viruses (Table 1). The RT-PCR assay for the fimoviruses could not distinguish them from each other since single infection of either one was not available in this study. In contrast to these viruses, the badnavirus, the marafivirus, and TPNRBV were not consistently associated with any visible symptoms.

**Table 1:** PCR and RT-PCR analysis of viruses (except betaflexiviruses) in *C. japonica* trees collected in three independent locations from the Jiangxi and Chongqing provinces.

**Tree**	**Symptom**	**CaCRSVs^g^**	**CaCRSVs^h^**	**CaCDaV**	**CaYRSV**	**TPNRBV**	**CaBaV**	**CaMaV**
JX1^a^	Chlorotic ringspot	+^d^	+	–^e^	–	–	+	–
JX2^f^	Chlorotic ringspot	+	+	–	–	–	–	–
JX3	Chlorotic ringspot	+	+	–	–	–	–	–
JX4	Chlorotic ringspot	+	+	–	–	–	+	–
JX5	Chlorotic ringspot	+	+	–	–	–	–	–
JX6	Chlorotic ringspot	+	+	–	–	–	–	–
JX7	Chlorotic ringspot	+	+	–	–	–	–	–
JX8	Chlorotic ringspot	+	+	–	–	–	–	–
JX9	Non-symptomatic	–	–	–	–	–	–	–
CRI1^b^	Malformation, mosaic	–	–	+	–	–	–	–
CRI2	Malformation, mosaic	–	–	+	–	–	–	–
CRI3	Asymptomatic	–	–	+	–	–	–	–
SWU1^c^	Malformation, mosaic	–	–	+	–	–	–	+
SWU2	Yellowing, mosaic	–	–	+	+	–	–	–
SWU3	Mosaic	–	–	+	+	–	–	–
SWU4	Yellow ringspot	–	–	–	+	+	–	–
SWU5	Yellowing, malformation	–	–	+	+	+	–	–
SWU6	Malformation, mosaic	–	–	+	–	+	–	–
SWU7	Malformation, mosaic	–	–	+	–	–	–	–
SWU8	Yellowing, malformation	–	–	+	+	+	–	–
SWU9	Yellow ringspot, malformation	–	–	+	+	+	–	–
SWU10	Malformation	–	–	+	+	+	–	–
SWU11	Yellowing	–	–	–	+	+	–	–
SWU12	Yellow spot	–	–	+	+	+	–	–
SWU13	Yellow ringspot	–	–	–	+	+	–	–
SWU14	Mosaic, chlorotic mottle	–	–	+	+	–	–	–
SWU15	Malformation	–	–	+	–	+	–	–
SWU16	Malformation	–	–	+	–	–	–	–
SWU17	Yellow ringspot, malformation	–	–	+	+	–	–	–
SWU18	Asymptomatic	–	–	+	+	–	–	–
SWU19	Malformation, mosaic	–	–	+	+	–	–	–
SWU20	Yellow ringspot	–	–	–	+	–	–	–
SWU21	Yellow ringspot	–	–	+	+	–	–	–
SWU22	Yellow ringspot	–	–	+	+	–	–	–
SWU23	Asymptomatic	–	–	+	–	–	–	–
SWU24	Yellow ringspot, malformation	–	–	+	+	–	–	–
SWU25	Malformation	–	–	+	+	–	–	–

a *JX trees from the Jiangxi (JX) province*.

b *CRI trees from Citrus Research Institute (CRI) in Chongqing province*.

c *SWU trees from Southwest University (SWU) in Chongqing province*.

d *Positive to a virus (+)*.

e *Negative to a virus (–)*.

f *Trees underlined showing a strong correlation of symptoms to a virus*.

g *Detection of RNA1-C3732*.

h *Detection of RNA1-C3473*.

From the perspective of a viral population (Table S4), the viral copy numbers of the fimoviruses were 23 times greater than that of the badnavirus in the SC-JX group. The reads of CaCDaV accounted for around 0.54% of the total reads of the SC-CRI group, which was much higher than those (<0.12%) of the other viruses. The copy number of the idaeovirus in the SC-L18 was 8 times higher than that of TPNRBV. These findings further suggested that the fimoviruses, the idaeovirus, and CaCDaV were associated with the symptom expressions in the host trees.

Based on the collective analysis of the presented data, the new viruses were provisionally named Camellia chlorotic ringspot viruses (CaCRSVs, the fimoviruses), Camellia yellow ringspot virus (CaYRSV, the idaeovirus), Camellia-associated badnavirus (CaBaV), and Camellia-associated marafivirus (CaMaV).

## Discussion

The foliar symptoms that were observed on *C. japonica* in this study resemble those that have been previously reported (Milbrath and McWhorter, 1946; Hildebrand, 1954; Ahlawat and Sardar, 1973; Gailhofer et al., 1988), but were more variable and complicated, especially the ringspot-associated symptoms occurring on either the same or different trees. These were yellow and chlorotic ringspots or spots with a diameter reaching the millimeter or centimeter levels (Figures 1B–D,I–L). For each type of the symptoms, it is important to explore how many viruses may be involved in development of the symptom and whether the culprit of each symptom is a sole virus or multiple viruses. To address these issues, samples from the *C. japonica* plants displaying different symptoms were analyzed by NGS coupled with homology-based method using BLAST programs which have been widely utilized for virus discovery (Wu Q. et al., 2015).

The NGS techniques have been used to explode in the discovery of new viral species associated with plant diseases (Adams et al., 2009; Hadidi et al., 2016). Experimental evidence from metagenomics based on NGS has revealed the natural biodiversity of plant viruses (Roossinck, 2011; Roossinck et al., 2015). A cryptic virus kingdom has yet to be explored since the research emphasis still largely focuses on the cultivated crops (Khoshbakht and Hammer, 2008), beyond which there are plentiful plant species distributed over the world (Pimm and Joppa, 2015). In this study, the NGS analyses of the ornamental camellias revealed the presence of the viruses related to the genus *Idaeovirus* and the families *Betaflexiviridae, Caulimoviridae, Fimoviridae, Geminiviridae, Kitaviridae*, and *Tymoviridae* (Adams et al., 2011; Dreher et al., 2011; Geering and Hull, 2011; MacFarlane, 2011; Zerbini et al., 2017; Elbeaino et al., 2018; Walker et al., 2019). The identification of ASGV and TPNRBV which are the known viruses infecting other economically important crops (Hao et al., 2018; Liu Q. et al., 2019) hinted at the potential roles of the infected *C. japonica* trees as viral reservoirs. Based on informatic analyses of the genomic features and phylogeny, the five new viruses were proposed to be new members of the demarcated taxa or even of a novel taxon (CCRaVs). These data indicated a rich diversity of viruses infecting the *C. japonica* plants.

The new fimoviruses (CCRaVs) infecting the camellias were validated to be consistent in genome architectures with the related emaraviruses infecting other plant species. The genomic RNA components of CCRaVs are likely to be at least pentapartite. RNA recombination, reassortment, and gene duplication that increase sequence variation or genome segmentation would contribute to the uncertainty in the acquisition of definite full genomes of emaraviruses (Tatineni et al., 2014; Di Bello et al., 2015; Lu et al., 2015; Patil et al., 2017; Yang C. et al., 2019). Until recently, two novel RNA segments of an emaravirus, European mountain ash ringspot-associated virus, were sequenced, in additional to the four known genomic RNAs (von Bargen et al., 2019). For CCRaVs, it is possible the additional viral RNA segments that are highly divergent from sequences of the available emaraviruses are present, and thus, they are undetectable in database-backed homology annotation.

Like other woody plants, ornamental camellias are connaturally perennial, which facilitates virus-plant symbiosis and symbiogenesis (Roossinck, 2008). In parallel to being limited to a single plant, viruses are capable of being transmitted from one plant to another in nature through vegetative propagation and vectors that play vital roles in the long-distance virus movement and increase the influence of the environmental changes exerted upon the course of virus diversification (Elena et al., 2014; Lefeuvre et al., 2019). Most of the extant viruses related to those identified from camellias in this study have specific vectors for their dispersals. For instance, badnaviruses, betaflexiviruses, and marafiviruses are transmitted by insect species infesting plants (Adams et al., 2011; Dreher et al., 2011; Bhat et al., 2016). Some phytophagous mites also contribute to the spread of emaraviruses and blunerviruses (Tatineni et al., 2014; Walker et al., 2019). For idaeoviruses, pollens transmission might be an effective way to transmit them (Bulger et al., 1990).

It is interesting that betaflexiviruses were detected in all the six sequenced sample groups independently of the geographic locations, while other viruses were not, suggesting there might be a long-term co-evolution of the betaflexiviruses with *C. japonica*. Given the possibility of natural transmission of these viruses, it is still not known whether *C. japonica* or other plants are the original hosts. Moreover, the global transport of massive plant materials and the unknown state of the plants carrying viruses make it difficult to trace the time when viruses of the source plant began to spread to native species or native viruses began to infect these plants.

Infections of multiple viruses in the same plant may not cause pathogenic effects on plants (Büttner et al., 2015), probably due to the balance kept among viruses or the convergent evolution of viruses toward mild interactions with the host (Roossinck, 2008). Otherwise, viral sequence variation, host genetic background, and environments all play generic roles in symptom development of plant viruses. These could be invoked to explain why the HC1 sample infected with many betaflexiviruses was asymptomatic, whereas the camellias infected with some of them showed some ringspot symptoms (Liu H. et al., 2019). It is also reasonable to characterize CaCDaV, CaCRSVs, and CaYRSV as symptom-associated, whereas TPNRBV, CaBaV, and CaMaV are not. However, the investigations must be widened to more regions and infected plants for confirmation.

In a natural setting, mixed infection of different viruses in a plant is the rule rather than the exception (Elena et al., 2014). To create a variety of flowers in a single *C. japonica* plant, scions of different origins are usually grafted by gardeners onto the same tree. This horticultural practice, as opposed to natural events, is considered as one of the sources for the viral coinfections. With respect to a strict Koch's rule required to support the opinion, transmission trials of the viruses infecting camellias are on the way.

In conclusion, this virome analysis of the *C. japonica* trees provides basic information of some viruses associated with the symptoms observed in this study and for evaluating the potential risk and management of the known or new viruses. The symptoms sporadically distributed among branches or/and plants were virus-related rather than derived from the *C. japonica* plants themselves.

## Data Availability Statement

The sequence information generated in this study can be retrieved from the NCBI database GenBank at CaMaV (MT036048), CaBaV (MT036049), CaCRSVs (MT040095–MT040104), and CaYRSV (MT036046 and MT036047).

## Author Contributions

MC conceived and designed the experiments. SZ, LY, and XT collected the samples and conducted the experiments. MC, LM, and SZ analyzed data. CZ, MC, RL, and SZ discussed the results and drafted and revised the manuscript. All authors read and approved the final draft of the manuscript.

## Conflict of Interest

The authors declare that the research was conducted in the absence of any commercial or financial relationships that could be construed as a potential conflict of interest.

## Supplementary Material

Figure S1 The sequencing strategies of the viral genomes. Black, red, and blue lines represent the viral full-length genome, contigs, and overlapping fragments amplified by PCR and RT-PCR, respectively. The contigs of the idaeovirus (CaYRSV) were derived from SC-L16, SC-L17, and SC-L18 sample sets.

Figure S2 Amino acid sequence alignment of two putative movement proteins (MP) of the fimoviruses (CaCRSVs) using the PROMALS3D. The 30K-MP domain is indicated by green bars. Alpha-helixes and beta-strands are indicated by red and blue backgrounds, respectively. The conserved catalytic amino acid residue Asp (D) appears in the red box.

Figure S3 The RNA reads (2,128) mapped in the viral genome of the badnavirus (CaBaV). The highest read was 85. The gray arrows represent ORFs.

Table S1 Next-generation sequencing analysis of six sample sets of 10 specimens collected from *C. japonica* plants grown in the Jiangxi and Chongqing provinces.

Table S2 List of primers used in this study.

Table S3 Pairwise aa sequence identity (%) shared between the fimoviruses (CaCRSVs) and representative emaraviruses at proteins encoded by RNA1 to RNA5. ^a^CaCRSVs, Camellia chlorotic ringspot viruses; AcEV-2, Actinidia emaravirus 2; AcCRaV, Actinidia chlorosis ringspot-associated virus; AsMaV, aspen mosaic-associated virus; BLMaV, blackberry leaf mottle associated virus; EMARaV, European mountain ash ringspot-associated virus; FMV, fig mosaic virus; HPWMoV, high plains wheat mosaic virus; JYMaV, jujube yellow mottle-associated virus; PiVB, Pistacia emaravirus; PPSMV and PPSMV-2, pigeonpea sterility mosaic virus and -2; PVWBV, palo verde witches broom virus; RLBV, raspberry leaf blotch virus; RRV, rose rosette virus; RYRaV, redbud yellow ringspot-associated virus; TiRSaV, ti ringspot-associated virus. ^b^na, not available.

Table S4 Virome analysis of the viruses (except the betaflexiviruses) identified from the symptomatic *C. japonica* plants that were sequenced.
