# Supplementary figures and images for: Virome of Camellia japonica: Discovery of and Molecular Characterization of New Viruses of Different Taxa in Camellias
Source: Front Microbiol. 2020 May 15;11:945. doi: 10.3389/fmicb.2020.00945 (PMC7243478; doi:10.3389/fmicb.2020.00945)

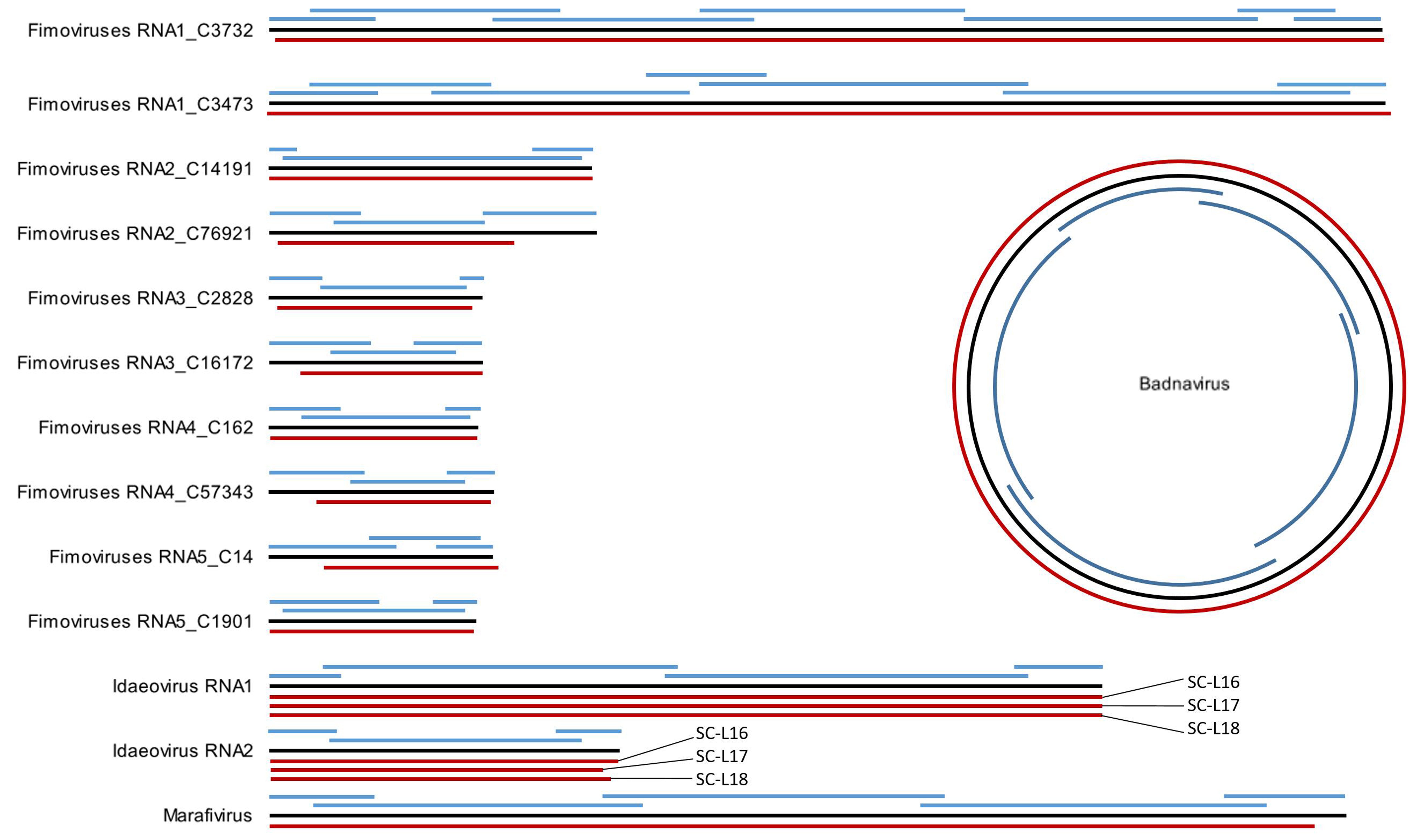

Supplement: Figure S1 — The sequencing strategies of the viral genomes. Black, red, and blue lines represent the viral full-length genome, contigs, and overlapping fragments amplified by PCR and RT-PCR, respectively. The contigs of the idaeovirus (CaYRSV) were derived from SC-L16, SC-L17, and SC-L18 sample sets. [file Image_1.JPEG]

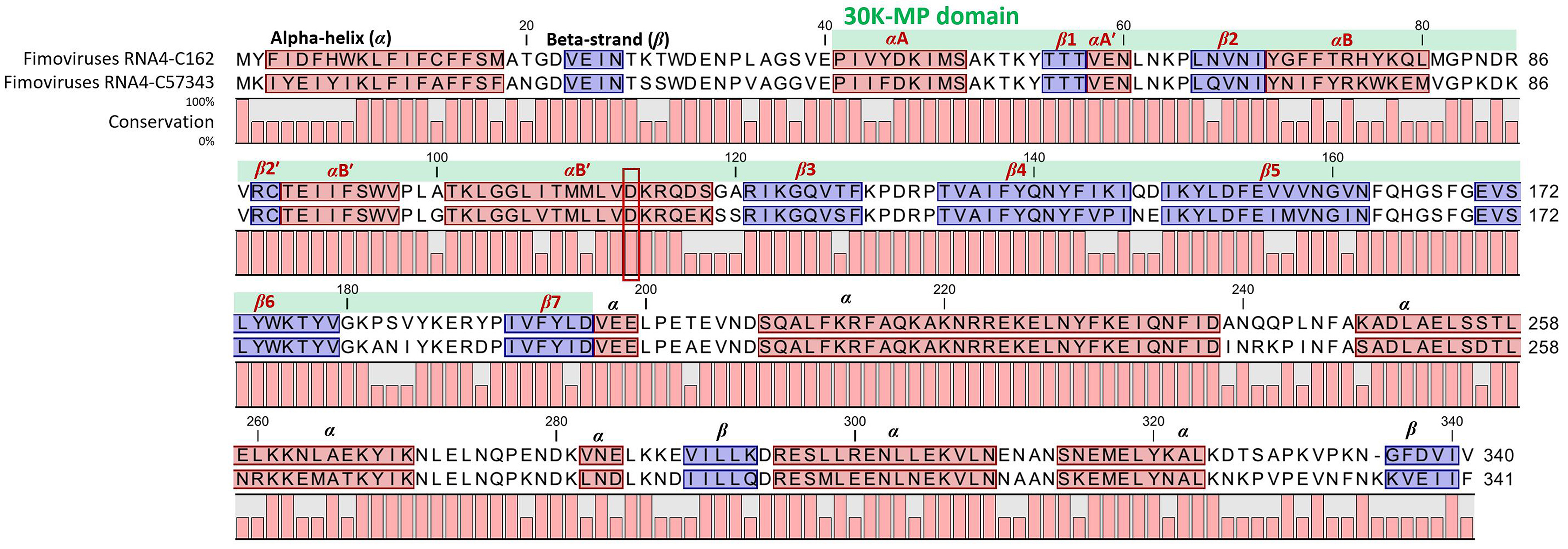

Supplement: Figure S2 — Amino acid sequence alignment of two putative movement proteins (MP) of the fimoviruses (CaCRSVs) using the PROMALS3D. The 30K-MP domain is indicated by green bars. Alpha-helixes and beta-strands are indicated by red and blue backgrounds, respectively. The conserved catalytic amino acid residue Asp (D) appears in the red box. [file Image_2.JPEG]

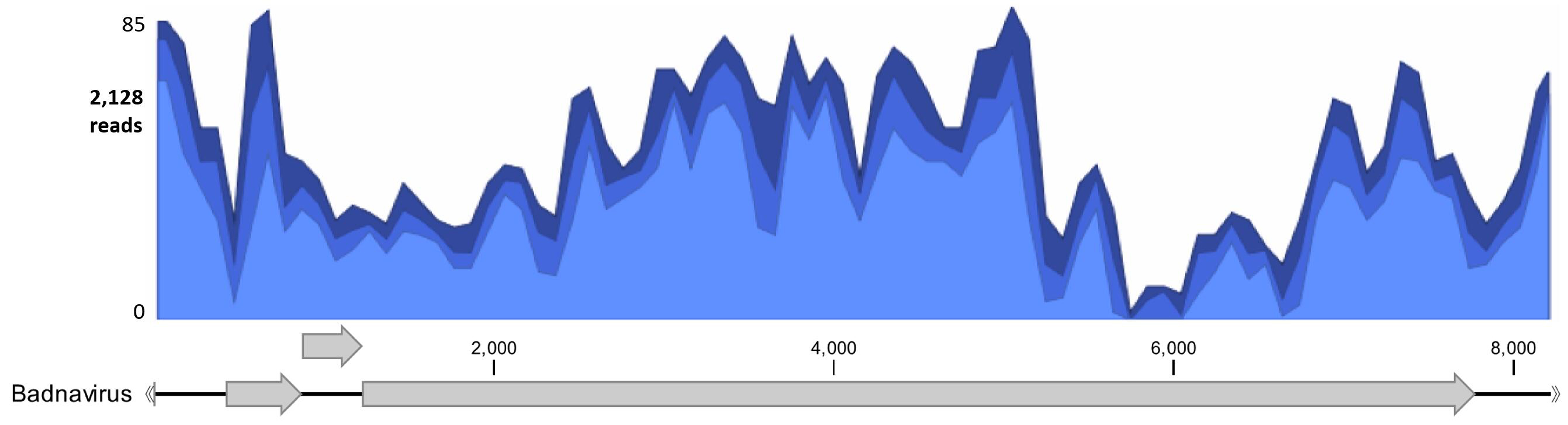

Supplement: Figure S3 — The RNA reads (2,128) mapped in the viral genome of the badnavirus (CaBaV). The highest read was 85. The gray arrows represent ORFs. [file Image_3.JPEG]
