# Supplementary material for: Virome of Camellia japonica: Discovery of and Molecular Characterization of New Viruses of Different Taxa in Camellias
Source: Front Microbiol. 2020 May 15;11:945. doi: 10.3389/fmicb.2020.00945 (PMC7243478; doi:10.3389/fmicb.2020.00945)
Supplement: Table S1 — Next-generation sequencing analysis of six sample sets of 10 specimens collected from C. japonica plants grown in the Jiangxi and Chongqing provinces. [file Data_Sheet_1.docx]

| **Sample set** | **Tree constitute** | **No. of clean reads** | **No. of candidate non-host clean reads** | **No. of contigs** | **Contigs length (nt)** | **Contigs-related viruses** | **No. of viral contigs** |
| --- | --- | --- | --- | --- | --- | --- | --- |
| SC-HC | HC1 | 68,315,102 (9.54 G) | 5,622,275 (8.23%) | 34,687 | 200–8,447 | betaflexiviruses | 16 |
| SC-JX | JX1 | 77,299,188 (10.8 G) | 3,100,900 (4.01%) | 24,491 | 200–7,447 | betaflexiviruses  fimoviruses  badnavirus | 3 |
|  |  |  |  |  |  |  | 10 |
|  |  |  |  |  |  |  | 1 |
| SC-CRI | CRI1, CRI2 | 88,826,086 (11.35 G) | 4,862,713 (5.47%) | 26,390 | 200–8,702 | betaflexiviruses geminivirus | 12 |
|  |  |  |  |  |  |  | 1 |
| SC-L16 | SC11 | 77,642,166 (10.84 G) | 3,352,543 (4.32%) | 15,876 | 200–8,750 | betaflexiviruses  idaeovirus  blunervirus | 7 |
|  |  |  |  |  |  |  | 2 |
|  |  |  |  |  |  |  | 4 |
| SC-L17 | SC1, SC14 | 71,377,718 (9.97 G) | 3,643,040 (5.1%) | 26,724 | 200–8,789 | betaflexiviruses  geminivirus  marafivirus idaeovirus | 11 |
|  |  |  |  |  |  |  | 1 |
|  |  |  |  |  |  |  | 1 |
|  |  |  |  |  |  |  | 2 |
| SC-L18 | SC4, SC13, SC20 | 77,832,684 (10.87 G) | 3,283,947 (4.22%) | 13,583 | 200–8,719 | betaflexiviruses  idaeovirus  blunervirus | 10 |
|  |  |  |  |  |  |  | 2 |
|  |  |  |  |  |  |  | 4 |

**Table S1** Next-generation sequencing analysis of six sample sets of 10 specimens collected from *C. japonica* plants grown in the Jiangxi and Chongqing provinces.

**Table S2**. List of primers used in this study.

| **Contigs-specific primers** | | **Sequence (5' to 3')** | **Base (nt)** | **Size (bp)** | |
| --- | --- | --- | --- | --- | --- |
| Marafivirus | M287F | AGCCACAGGACCTCTCACGTAC | 22 | | 2087 |
|  | M2373R | GGGGATCTGGGAACGAGAACTC | 22 | |  |
|  | M2137F | AAGTCTCCCCTCAGATTCCGCT | 22 | | 2104 |
|  | M4326R | TCGTCTCCGGCAAGAAGTGTGT | 22 | |  |
|  | M4166F | GGAAGACCCCTACTCTCTCGCT | 22 | | 2212 |
|  | M6377R | GGTGATGGTTTTCAGAGTCGCT | 22 | |  |
| Fimoviruses | F258F | CCTTTGTAATGTCATCAGCATAACT | 25 | | 1603 |
| RNA1-C3732 | F1860R | AGTCAGTCTGTATTAACACCATCAC | 25 | |  |
|  | F1426F | ATACATTCCAATCCTTTCAAGCCAG | 25 | | 1692 |
|  | F3117R | GAAAACCCTGAGAACACTGGTAAAT | 25 | |  |
|  | F2756F | CCTTCTGATGGGTTTTTCTTTGTTG | 25 | | 2207 |
|  | F4452R | TTATGGATTCTCTTTCTGGGCTATG | 25 | |  |
|  | F4438F | CATAGCCCAGAAAGAGAATCC | 21 | | 1891 |
|  | F6329R | TCAGTGATGACAATGTGGC | 19 | |  |
|  | F6194F | TCAAGGTCTTTTATCATGACATCCC | 25 | | 631 |
|  | F6824R | ATGAATTTATAAGCACTGAGCCACA | 25 | |  |
| Fimoviruses | F257F | TGTGATTGATTGATCTACGGGTATA | 25 | | 1164 |
| RNA1-C3473 | F1418R | GGGAAAGATATATTTGTAACTGGGA | 25 | |  |
|  | F1037F | CTCATTTGCCTTTCTGCC | 18 | | 1657 |
|  | F2694R | GATGTTTGCCTTACTACACTGC | 22 | |  |
|  | F2412F | ACTGGTGCGATAGTTATTAGTGATG | 25 | | 771 |
|  | F3182R | GTTGCTTTGGTCTAGAAACAATATG | 25 | |  |
|  | F2749F | CAATCAGCATTTCAGAGACACCTAA | 25 | | 2114 |
|  | F4862R | ATTCAATCCAACCCCCAAAACTATC | 25 | |  |
|  | F4694F | AACCGTTGCTTCAAAACCTTTATCT | 25 | | 2232 |
|  | F6925R | GGTCAAAAGAAGACTGAATTGGCTA | 25 | |  |
| Fimoviruses | F81F | TGCTCACATTACGAAGCTCA | 20 | | 1912 |
| RNA2-C14191 | F1992R | CTTCTGGCTTTGTGCGTT | 18 | |  |
| Fimoviruses | F410F | TTGAGCTCATCTTGGTCGATTATAT | 25 | | 958 |
| RNA2-C76921 | F1367R | GTGAGAATATGACCAAGCTGAG | 22 | |  |
| Fimoviruses | F322F | TCTGCCTATTTTTCATCAAGGACTT | 25 | | 939 |
| RNA3-C2828 | F1260R | ACATGTCGAGTTCTAGTGGAAGCAA | 25 | |  |
| Fimoviruses | F396F | TTCCGATGGTAAACCTGATTGA | 22 | | 797 |
| RNA3-C16172 | F1192R | CCAAAGTGCCTGTAAAACATAGC | 23 | |  |
| Fimoviruses | F203F | GAACTTTTGGTGCTGATGTG | 22 | | 1078 |
| RNA4-C162 | F1280R | AGTCATTTTGGATTGAGTGGATTT | 24 | |  |
| Fimoviruses | F521F | TGGCTGGTTTAATTCAAGGT | 20 | | 730 |
| RNA4-C57343 | F1250R | AGTTGCAGGTGGTGTTGAG | 19 | |  |
| Fimoviruses | F641F | ATCTATAAGTCTGGCTTCCAACTC | 24 | | 711 |
| RNA5-C14 | F1351R | AAGGTTTTCATACGTTGACTTGTTG | 25 | |  |
| Fimoviruses | F93F | GCGATTCAAGCTGTTCTCA | 19 | | 886 |
| RNA5-C1901 | F1250R | AGCCTTGCATACGAGTTGAT | 20 | |  |
| Idaeovirus | R341F | CTGTAAGGCATTTCGTGGTAGG | 22 | | 2270 |
| RNA1 | R2610R | CCAAACCGGAGTATCACCATCA | 22 | |  |
|  | R2535F | TACCGAATTGAACCATGAGGCT | 22 | | 2308 |
|  | R4842R | TGCCAGTGGACCCAACTCAATA | 22 | |  |
| Idaeovirus | R364F | TCGAGAGGCAAAAGTAAACAGC | 22 | | 1624 |
| RNA2 | R1987R | AATTCGAAGAACTTCCGTCCGG | 22 | |  |
| Badnavirus | B129 F | AGTTATTGTATGTCAACCAGGGC | 23 | | 1581 |
|  | B1709R | TATCTCTCAGGACTAAGTGTGCC | 23 | |  |
|  | B1564F | TGACCTGCCATTCATAGCG | 19 | | 1969 |
|  | B3532R | GATTGTCTCCCAACGAACG | 19 | |  |
|  | B3499F | GTTTGATGAAGTGTTCGTTCG | 21 | | 1956 |
|  | B5454R | TTGATTTGCTGCTGCGTC | 18 | |  |
|  | B5333F | AGGATACAGATGATAATCGGCTG | 23 | | 2068 |
|  | B7400R | TGAGGATAGAGATAAGGTTCACG | 23 | |  |
|  | B7336F | CGAGGCAGAGGAAAGTATTGAA | 22 | | 1139 |
|  | B253R | TGCGTTCTTACGTCTCTTGGTT | 22 | |  |
| **RACE primers** | | **Sequence (5' to 3')** | **Base (nt)** | | **Size (bp)** |
| Marafivirus | M5R | AGGAAGGGCTGGAGGTTTTGAGG | 23 | | 610 |
|  | M3R | CTCTCAACACACCTCCAGCTCTC | 23 | | 764 |
| Fimoviruses | F5R3732 | AAAAGGGATGTAAATGGACCTATAT | 25 | | 708 |
| RNA1-C3732 | F3R3732 | TGCATGATGCTCAATGACATTCTTA | 25 | | 590 |
| Fimoviruses | F5R3473 | ATAAAAGACACTAATCGGCTCATCC | 25 | | 706 |
| RNA1-C3473 | F3R3473 | AGCTCAGATGTGTTCGGTATTGTAT | 25 | | 707 |
| Fimoviruses | F5R14191 | AGCTTTCATTGTTTTCACACCATCT | 25 | | 200 |
| RNA2-C14191 | F3R14191 | CATATTGGTTGAAAAACTGTGACAC | 25 | | 414 |
| Fimoviruses | F5R76921 | TGTGAACTTCGAGCTAAAATTGATG | 25 | | 588 |
| RNA2-C76921 | F3R76921 | GGTCATATTCTCACCAACCTTTTCT | 25 | | 750 |
| Fimoviruses | F5R2828 | TCCTTGATGAAAAATAGGCAGATAG | 25 | | 343 |
| RNA3-C2828 | F3R2828 | TTTTTGCCAATGACTATCTTGCTTC | 25 | | 156 |
| Fimoviruses | F5R16172 | TTTGAATCATTCCCTGAAGAAGTTG | 25 | | 656 |
| RNA3-C16172 | F3R16172 | CTCTATCAATTGCCTCTTTGCTGGA | 25 | | 432 |
| Fimoviruses | F5R162 | AAGCAGATTTAGCAGAGTTGTCAAG | 25 | | 455 |
| RNA4-C162 | F3R162 | GATTTTCATCCCAGGTTTTAGTGTT | 25 | | 254 |
| Fimoviruses | F5R57343 | TTTGCATCAGCAGACTTAGCAGAGC | 25 | | 616 |
| RNA4-C57343 | F3R57343 | GTCCAACCATTTCCTTCCACTTTCT | 25 | | 336 |
| Fimoviruses | F5R14 | ATGCACAATTTGGATTTGAAACTGG | 25 | | 818 |
| RNA5-C14 | F3R14 | TTTACCTATTTGTCTCCCAATTCCA | 25 | | 365 |
| Fimoviruses | F5R1901 | GTTTCGAAACTGATATTGGTTGTGA | 25 | | 706 |
| RNA5-C1901 | F3R1901 | TTCCATTAGGATTCATACCATTTGC | 25 | | 312 |
| Idaeovirus | I5R1 | GGCTACCGTCTTAGAGAAATACCAG | 25 | | 447 |
| RNA1 | I3R1 | TTGGAGGGGATGATTCACTTATTTG | 25 | | 545 |
| Idaeovirus | I5R2 | CAAGATTGAAGATACCTCGTGATGG | 25 | | 424 |
| RNA2 | I3R2 | ATAATAGTGGACTGCAAAGTGACCG | 25 | | 416 |
| **Detection primers** | | **Sequence (5' to 3')** | **Base (nt)** | | **Size (bp)** |
| Geminivirus | CP1-F | AACAGTTTACCACGACGGAC | 20 | | 537 |
|  | CP1-R | TGGCTAAGATTGCCTTCATC | 20 | |  |
| Blunervirus | TPNRBV1-F | GCCCTGACAA CGCAAAAGAA CTGATG | 26 | | 535 |
|  | TPNRBV1-R | GTGACGGGAT ATTTTTGGAC GACTGT | 26 | |  |
| Marafivirus | MD1-F | CCATCATACCCTGGATTGC | 19 | | 491 |
|  | MD1-R | CGTAGTAGGCGGTTTCAGTC | 20 | |  |
| Fimoviruses | FD1F | GGATTCTCTTTCTGGGCTATG | 21 | | 776 |
| RNA1-C3732 | FD1R | GGTGTTGTTATTGCTTCCTCTC | 22 | |  |
| Fimoviruses | FD2F | CGGATAAATCATTGTTGGAGTC | 22 | | 783 |
| RNA1-C3473 | FD2R | CATCAGCAGTGTAGTAAGGCAA | 22 | |  |
| Idaeovirus | ID1-F | AATGATAAGGACTACTGGGTGC | 22 | | 316 |
|  | ID1-R | TTTCGTCGGTCACTTTGC | 18 | |  |
| Badnavirus | BD1-F | CAAGCAAGAGTCGTCATCG | 19 | | 750 |
|  | BD1-R | TTCAGCATTCATCCGTCG | 18 | |  |

**Table S3** Pairwise aa sequence identity (%) shared between the fimoviruses (CaCRSVs) and representative emaraviruses at proteins encoded by RNA1 to RNA5.

| **Virus ^1^/RNA** | **RNA1-C3732** | **RNA1-C3473** | **RNA2-C14191** | **RNA2-C76921** | **RNA3-C2828** | **RNA3-C16172** | **RNA4-C162** | **RNA4-C57343** | **RNA5-C14 ^2^** | **RNA5-C1901** |
| --- | --- | --- | --- | --- | --- | --- | --- | --- | --- | --- |
| CaCRSVs | 58.08 | | 44.28 | | 43.65 | | 74.78 | | 53.88 | |
| AcEV-2 | 26.89 | 26.53 | 19.91 | 18.76 | 18.00 | 15.52 | 13.08 | 13.30 | 3.58 | 3.98 |
| AcCRaV | 27.42 | 27.83 | 17.56 | 18.17 | 14.62 | 15.00 | 14.47 | 14.21 | 4.11 | 3.81 |
| AsMaV | 27.22 | 26.82 | 18.70 | 17.77 | 16.29 | 14.08 | 13.08 | 12.28 | 3.72 | 4.60 |
| BLMaV | 27.25 | 27.01 | 19.83 | 17.42 | 16.09 | 14.41 | 14.29 | 13.99 | 3.99 | 5.85 |
| EMARaV | 27.22 | 27.17 | 19.60 | 18.70 | 16.23 | 16.28 | 13.08 | 12.79 | 3.59 | 2.40 |
| FMV | 26.79 | 27.13 | 19.28 | 18.40 | 17.77 | 16.43 | 17.71 | 17.45 | 3.53 | 3.54 |
| HPWMoV | 24.22 | 25.15 | 20.06 | 19.36 | 15.88 | 14.54 | 18.75 | 20.83 | 20.94 | 21.25 |
| JYMaV | 26.57 | 26.16 | 19.50 | 17.74 | 17.93 | 14.76 | 14.10 | 13.30 | 6.69 | 5.70 |
| PiVB | 27.81 | 27.69 | 19.13 | 16.60 | 18.34 | 16.62 | 16.11 | 14.80 | 5.33 | 5.34 |
| PPSMV | 26.65 | 27.66 | 19.28 | 17.08 | 17.06 | 17.11 | 12.82 | 13.04 | 3.76 | 3.76 |
| PPSMV-2 | 26.50 | 26.59 | 20.83 | 16.74 | 17.24 | 15.85 | 18.32 | 19.63 | 4.15 | 3.96 |
| PVWBV | 25.55 | 25.22 | 18.57 | 18.12 | 11.59 | 10.69 | 21.70 | 21.39 | na | na |
| RLBV | 27.13 | 26.75 | 18.74 | 16.80 | 15.60 | 12.42 | 12.31 | 11.76 | 6.60 | 6.31 |
| RRV | 26.30 | 26.58 | 19.83 | 17.59 | 16.00 | 13.43 | 16.11 | 14.54 | 3.40 | 3.88 |
| RYRaV | 27.91 | 27.86 | 19.45 | 19.20 | 13.33 | 13.99 | 18.30 | 18.52 | 4.11 | 3.81 |
| TiRSaV | 25.38 | 26.18 | 18.68 | 17.25 | 13.72 | 14.50 | 13.08 | 13.30 | 3.99 | 4.67 |

^1^ CaCRSVs, Camellia chlorotic ringspot viruses; AcEV-2, Actinidia emaravirus 2; AcCRaV, Actinidia chlorisis ringspot-associated virus; AsMaV, aspen mosaic-associated virus; BLMaV, blackberry leaf mottle associated virus; EMARaV, European mountain ash ringspot-associated virus; FMV, fig mosaic virus; HPWMoV, high plains wheat mosaic virus; JYMaV, jujube yellow mottle-associated virus; PiVB, Pistacia emaravirus; PPSMV and PPSMV-2, pigeonpea sterility mosaic virus and -2; PVWBV, palo verde witches broom virus; RLBV, raspberry leaf blotch virus; RRV, rose rosette virus; RYRaV, redbud yellow ringspot-associated virus; TiRSaV, ti ringspot-associated virus. ^2^ na, not available.

**Table S4** Virome analysis of the viruses (except the betaflexiviruses) identified from the symptomatic *C. japonica* plants that were sequenced.

| **Data** | **Virus** | **Genome** | **No. of viral reads** | **Viral copies** | **No. of viral reads in total** | **% of total clean reads** |
| --- | --- | --- | --- | --- | --- | --- |
| SC-JX | CaCRSVs | -ssRNA1-C3732 | 24,312 | 509 | 58,460 | 0.076 |
|  |  | -ssRNA1-C3473 | 1,129 | 24 |  |  |
|  |  | -ssRNA2-C14191 | 8,607 | 619 |  |  |
|  |  | -ssRNA2-C76921 | 137 | 10 |  |  |
|  |  | -ssRNA3-C2828 | 8,121 | 880 |  |  |
|  |  | -ssRNA3-C16172 | 311 | 34 |  |  |
|  |  | -ssRNA4-C162 | 6,685 | 736 |  |  |
|  |  | -ssRNA4-C57343 | 181 | 20 |  |  |
|  |  | -ssRNA5-C14 | 4,324 | 449 |  |  |
|  |  | -ssRNA5-C1901 | 4,643 | 518 |  |  |
|  | CaBaV | circle dsDNA | 2,128 | 38 | 2,128 | 0.0028 |
| SC-CRI | CaCDaV | circle ssDNA | 476,689 | 19,199 | 476,689 | 0.54 |
| SC-L16 | CaYRSV | +ssRNA1 | 7,521 | 211 | 16,948 | 0.022 |
|  |  | +ssRNA2 | 9,427 | 626 |  |  |
|  | TPNRBV | +ssRNA1 | 22,765 | 575 | 79,715 | 0.10 |
|  |  | +ssRNA2 | 25,421 | 921 |  |  |
|  |  | +ssRNA3 | 16,017 | 897 |  |  |
|  |  | +ssRNA4 | 15,512 | 1,258 |  |  |
| SC-L17 | CaCDaV | circle ssDNA | 562 | 23 | 562 | 0.00079 |
|  | CaMaV | +ssRNA | 84,566 | 1,833 | 84,566 | 0.12 |
|  | CaYRSV | +ssRNA1 | 2,292 | 64 | 11,494 | 0.016 |
|  |  | +ssRNA2 | 9,202 | 610 |  |  |
| SC-L18 | CaYRSV | +ssRNA1 | 13,906 | 389 | 40,920 | 0.052 |
|  |  | +ssRNA2 | 27,014 | 1,790 |  |  |
|  | TPNRBV | +ssRNA1 | 5,779 | 146 | 17,097 | 0.022 |
|  |  | +ssRNA2 | 5,506 | 199 |  |  |
|  |  | +ssRNA3 | 3,243 | 181 |  |  |
|  |  | +ssRNA4 | 2,551 | 206 |  |  |
